# Radiological cervical spine involvement in polyarticular idiopathic juvenile arthritis

**DOI:** 10.1186/1546-0096-9-S1-P121

**Published:** 2011-09-14

**Authors:** J Wipff, M Elhai, R Bazeli, A Feydy, A Kahan, C Job-Deslandre

**Affiliations:** 1 Rheumatology A, Paris Descartes University, Cochin Hospital, APHP, Paris, France; 2 Radiology B, Paris Descartes University, Cochin Hospital, APHP, Paris, France

## Background

Radiological involvement of the cervical spine has already been assessed in juvenile idiopathic arthritis (JIA) but most studies have been performed in symptomatic JIA and without differentiating subsets of JIA.

## Aim

To describe structural inflammatory cervical spine involvement in pJIA regardless of cervical symptoms and compare observed lesions to those seen in RA using a cross-sectional observational study.

## Patients and method

All consecutive pJIA followed in a transition program have been included in this study. Demographic and rheumatism characteristics were collected. Laboratory tests and standard radiographies of the cervical spine (antero-posterior, lateral view with flexion and extension and open mouth view), hands and feet were performed. A RA control group (<55years), matched for sex and disease duration, was recruited.

## Results

58 pJIA (48 females/10 males) and 59 RA (52/7) were included. Respectively, mean age was 23.6±10 years and 43.2±9.6 years and mean disease duration 13.14±11.14 and 12.17±7.1 years. 60% and 80% (p=0.02, OR 0.39[0.17-0.89]) were RF positive. Radiographies showed inflammatory lesions in 38/58 pJIA (66%) and 40/59 (68%) RA patients and respectively 3/56 and 2/59 patients underwent cervical surgery.

In pJIA with radiological lesions, the most frequent lesions were anterior atlantoaxial subluxation (35%), erosions of the odontoid process (34%) and atlanto-axial impaction (20%). Apophyseal joint ankylosis (AJA) was rare (10%) but involvement of the posterior cervical spine (AJA and apophyseal joint arthritis) was more frequent (42%).

Presence of radiographic cervical lesions was correlated in univariate analysis with a more severe disease phenotype: more erosive disease (hand and feet), higher number of biotherapies per patient (respectively 1.4±1.3 vs 0.5±0.5, p=0.003), and higher frequency of surgery (p=0.03, OR 7.74[0.92-65]) but not with RF status. Multivariate analysis confirmed only the association with the higher number of biotherapies per patients (p=0.04).

50% of pJIA with radiographic abnormalities had no clinical symptoms. In pJIA sub-group with cervical symptoms (n=24), patients needed more DMARDs and biotherapies per patient.

Finally, comparison between pJIA and RA for radiographic findings showed only differences for two structural features: hypotrophy of vertebrae (24% vs 7%) and AJA (10% vs 0%).

## Conclusion

Structural cervical spine involvement is a frequent manifestation in pJIA followed at adulthood and is correlated with a more severe disease. Radiologic assessment of cervical spine should be done systematically at onset and regularly during the course of the disease regardless of clinical symptoms. Cervical symptoms may be considered as a warning signal revealing presence of AJA.

